# Supplementary material for: Splice-Junction-Based Mapping of Alternative Isoforms in the Human Proteome
Source: Cell Rep. Author manuscript; Available in PMC 2020 Jan 15. (PMC6961840; doi:10.1016/j.celrep.2019.11.026)
Supplement: 2 [file NIHMS1546469-supplement-2.zip › DF1/PXD010154/Prostate/Prostate_7_SORBS1_MTNLDKDLSLCQTELEADLEK.pdf]

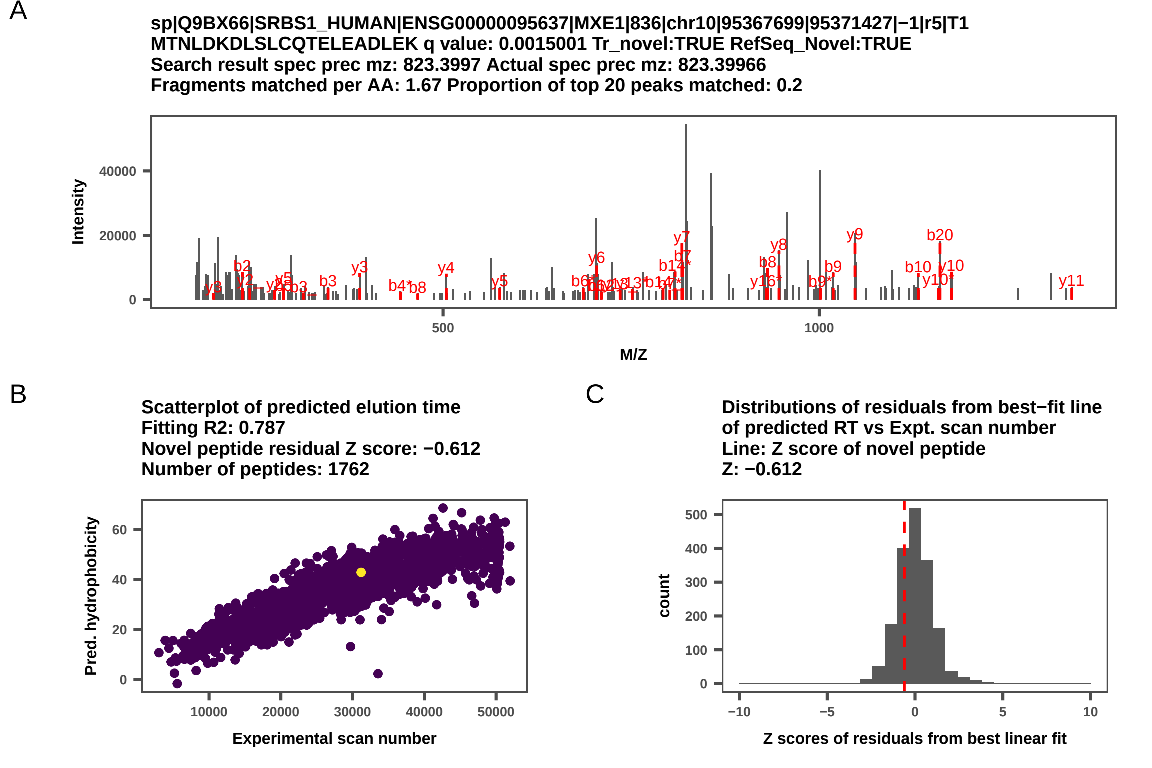
A
sp|Q9BX66|SRBS1_HUMAN|ENSG00000095637|MXE1|836|chr10|95367699|95371427|−1|r5|T1
MTNLDKDLSLCQTELEADLEK q value: 0.0015001 Tr_novel:TRUE RefSeq_Novel:TRUE
Search result spec prec mz: 823.3997 Actual spec prec mz: 823.39966
Fragments matched per AA: 1.67 Proportion of top 20 peaks matched: 0.2
Intensity
40000
20000
0
M/Z
500
1000
y3
b2
y2
y5
b3
y3
b4*
b8
y4
y5
y6
y7
b7
b14*
y8
b8
y16*
b9
b9*
y9
b10
b20
y10
y10*
y11
B
Scatterplot of predicted elution time
Fitting R2: 0.787
Novel peptide residual Z score: −0.612
Number of peptides: 1762
Pred. hydrophobicity
60
40
20
0
10000
20000
30000
40000
50000
Experimental scan number
C
Distributions of residuals from best−fit line
of predicted RT vs Expt. scan number
Line: Z score of novel peptide
Z: −0.612
count
500
400
300
200
100
0
−10
−5
0
5
10
Z scores of residuals from best linear fit
